# Supplementary material for: Antagonistic Activity of Oroxylin A against Fusarium graminearum and Its Inhibitory Effect on Zearalenone Production
Source: Toxins (Basel). 2023 Aug 31;15(9):535. doi: 10.3390/toxins15090535 (PMC10535041; doi:10.3390/toxins15090535)
Supplement: Supplementary file 1 [file toxins-15-00535-s001.zip › toxins-2558624-supplementary.pdf]

Supplementary Tables and Figures

Table S1. Tentative identification of Oroxylin A via PSE by UPLC-MS/MS.

Compound	Formula	Adducts [a]	△ppm [b]	Score [c]
Oroxylin A	$C_{16}H_{12}O_5$	[M-H]	1.11	94.5

[a] Compound was detected in negative ([M-H]) ion modes.
[b] Mass error of the theoretical and the experimental precise molecular weight.
[c] Compound was tentatively identified by matching their primary and secondary mass spectrums with the mzCloud library (https://www.mzcloud.org). Higher scores indicate a higher similarity.

Table S2. Differential metabolites identified between T1 and T0 and between T2 and T0

Number	Name	*p*-value [a]	FC [b]
T1 vs T0			
1	Aminoadipic acid	2.44×10^{-3}	2.21
2	Itaconic acid	7.62×10^{-3}	1.75
3	Citraconic acid	2.83×10^{-3}	1.57
4	*cis*-Aconitate	8.90×10^{-3}	1.56
5	*L*-Homoserine	2.51×10^{-2}	1.29
6	Nicotinamide ribotide	3.54×10^{-2}	0.72
7	*p*-Aminobenzoate	4.32×10^{-2}	0.72
8	Phosphorylcholine	2.50×10^{-2}	0.45
9	R5P (*D*-Ribose-5-phosphate)	3.65×10^{-3}	0.17
T2 vs T0			
1	Fumarate	1.30×10^{-2}	3.62
2	*L*-Homocysteic acid	1.30×10^{-2}	2.33
3	Ascorbic acid	4.71×10^{-2}	2.33
4	Shikimate	4.58×10^{-2}	2.21
5	Coenzyme A	3.78×10^{-2}	2.00
6	5-Methoxytryptophan	3.82×10^{-2}	0.68
7	Spermine	3.58×10^{-3}	0.58
8	R5P (*D*-Ribose-5-phosphate)	3.29×10^{-2}	0.32

[a] *P*-values were calculated using a Student's *t*-test.
[b] Fold change (FC) was calculated by the average value of T1 compared to that of T0, or T2 compared to that of T0. FC with a value larger than 1.1 indicates a higher level of the metabolite in T1 or T2; FC value lower than 0.9 indicates a lower level compared to T0.

Table S3. Primer sequences used for RT-PCR analysis

Gene	Primer sequences (5′→3′)	Product size (bp)
EF 1-α	F: GGCTTTCACCGACTACCCTCCTCT<br>R: ACTTCTCGACGGCCTTGATGACAC	91
ZEB1	F: AAATAATTTACCCGTTCTTCTGGGAACT<br>R: CTGAAACGGAGGTGTTGAGG	129
ZEB2	F: GGGATTAACCGCTGTGG<br>R: TAGGCATGCCCGAAACCGAAAGT	80

EF1-α: Elongation factor 1-α; *ZEB1:* Zearalenone biosynthesis protein 1; *ZEB2:* Zearalenone biosynthesis protein 2.

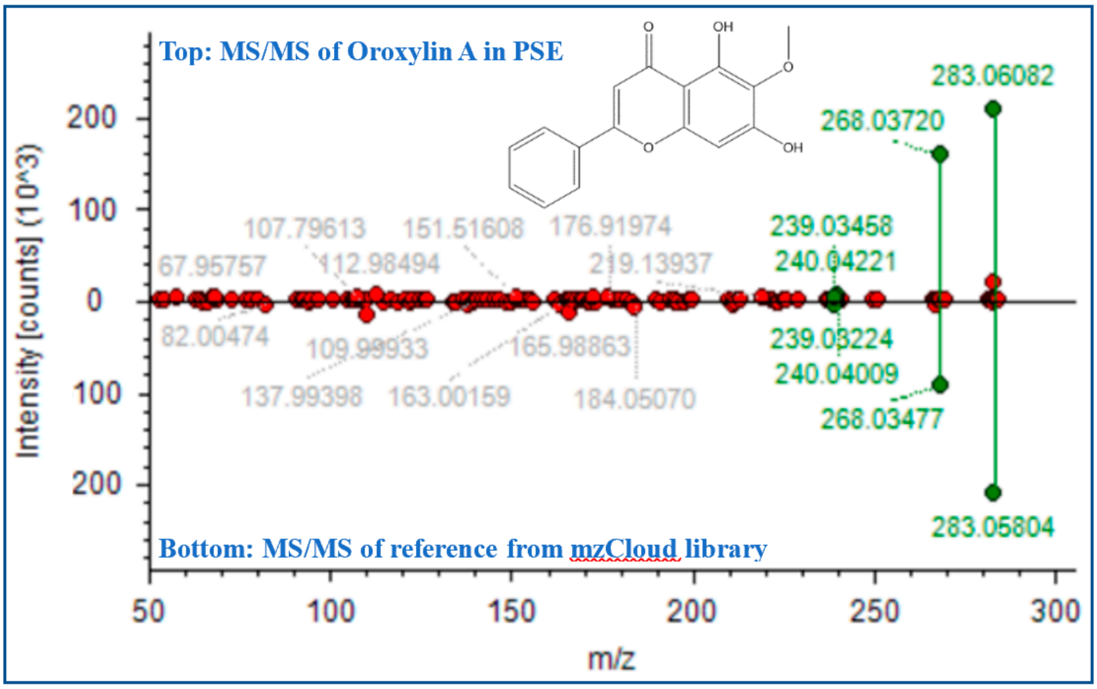

Figure S1 MS/MS spectra of Oroxylin A tentatively identified in PSE.
